# Exploring the Potential of Emerging Technologies to Meet the Care and Support Needs of Older People: A Delphi Survey

**DOI:** 10.3390/geriatrics6010019

**Published:** 2021-02-13

**Authors:** Sarah Abdi, Luc de Witte, Mark Hawley

**Affiliations:** Centre for Assistive Technology and Connected Healthcare, School of Health and Related Research, University of Sheffield, Sheffield S1 4DP, UK; sabdi1@sheffield.ac.uk (S.A.); l.p.dewitte@sheffield.ac.uk (L.d.W.)

**Keywords:** Delphi, artificial intelligence, voice activated devices, robotics, older people, care and support

## Abstract

Some emerging technologies have potential to address older people’s care and support needs. However, there is still a gap in the knowledge on the potential uses of these technologies in some care domains. Therefore, a two-round Delphi survey was conducted to establish a consensus of opinion from a group of health and social technology experts (*n* = 21) on the potential of 10 emerging technologies to meet older people’s needs in five care and support domains. Experts were also asked to provide reasons for their choices in free-text spaces. The consensus level was set at 70%. Free-text responses were analyzed using thematic analysis. Voice activated devices was the technology that reached experts consensus in all assessed care domains. Some technologies (e.g., Artificial intelligence (AI) enabled apps and wearables and Internet of things (IoT) enabled homes) also show potential to support basic self-care and access to healthcare needs of older people. However, most of the remaining technologies (e.g., robotics, exoskeletons, virtual and augmented reality (VR/AR)) face a range of technical and acceptability issues that may hinder their adoption by older people in the near future. Findings should encourage the R & D community to address some of the identified challenges to improve the adoption of emerging technologies by older people.

## 1. Introduction

Recent statistics in the United Kingdom (UK) estimated that around 22% of males and 31% of females aged over 65 years require care and support due to living with limiting long-term conditions [1]. Examples of support and care activities include taking medication, getting around and shopping [2]. The number of older people requiring care and support is also expected to increase by 25% in the year 2025 [3] and 67% in the year 2040 [4], raising a challenge to meet the increasing need for care and support from the older population. Additionally, many older people with care and support needs prefer to continue living in their homes as long as possible [5]. Supporting older people to continue living in their own homes and keeping them out of residential care is also a well-recognized priority to local authorities in the UK [6]. However, many concerns have been raised about the sustainability of the formal and informal care systems to meet older people’s care and support needs living at home [6,7]. The health and social care system, for instance, is facing financial pressures threatening their ability to meet increasing demands from an ageing population [6]. Family carers are also facing care related physical, mental and financial challenges that threaten their ability to sustain their caring responsibilities [7]. These challenges have, indeed, resulted in many older people not receiving the care and support required to continue living in their homes. For example, 55% of older people who have difficulty with an activity of daily living and 80% with mobility difficulty did not receive formal or informal support, according to a recent analysis from wave 7 of the English Longitudinal Study of Ageing (ELSA) [8]. Therefore, there is an urgent need to address this gap in care, given the negative impact unmet care needs have on older people’s health and wellbeing [9].

Technology offers a potential solution to address some of older people’s care needs. In recent years, a number of technologies have demonstrated a positive impact on older people’s physical and mental health and on their social lives [10,11,12,13,14]. For example, a review on the effectiveness of technologies on social isolation amongst older people reported positive results, mainly with telecare, video games, information communication technologies (ICT) and robotics [10]. Similarly, the potential of telehealth in reducing hospital readmission and improving the health outcomes of older people was documented several times in the last few years [13,14]. More recently, emerging technologies—technologies characterized by novelty, growth and potential socio-economic impact—have demonstrated some potential to support older people’s care needs at home [15,16,17,18]. These technologies build on recent advances and interdependencies between artificial intelligence (AI) and subset technologies (e.g., speech recognition), sensor technology, and advances in connectivity and computing (e.g., fifth-generation cellular wireless (5G) and edge computing) to offer new ways of interacting and communicating with technology [15,16]. For example, voice activated devices (e.g., Amazon’s Alexa and Google assistant) can support older people with basic care tasks such as medication reminders and providing information [15]. Similarly, Internet of Things (IoT) enabled homes can automate home experiences for older people through allowing various technologies to interact and communicate with each other [15]. However, it remains the case that the rate of adopting new technologies in the older population is lower than other age groups [19,20]. Some of the main reasons for this are lack of perceived value and positive impact of technology on older people’s quality of life as well as lack of confidence in their digital skills [19,21,22,23]. For example, a recent study reported that failure to identify essential uses of Alexa (a voice activated device) was one of the main reasons for abandoning its use by older people over time [24]. Therefore, there is arguably a need to further explore the potential of emerging technologies and highlight their potential uses and benefits for older people. In particular, it will be useful to explore the potential of these technologies in care domains considered as important areas of support for older people living at home. These include mobility, self-care and domestic life, social life and relationships, psychological support and access to healthcare [2].

Recently, a scoping review of grey literature identified a range of emerging technologies that could potentially address challenges related to these domains [15]. These technologies included self-driving vehicles, assistive autonomous robots, exoskeletons, AI-enabled mobile applications and wearables, new drug release mechanisms (e.g., DNA origami and digital pills), portable diagnostics, voice activated devices, virtual, augmented and mixed reality (VR/AR/MR) and IoT enabled homes. However, the review highlighted that there is still a gap in the knowledge on the potential uses of these technologies in some care domains such as social life and psychological support. Additionally, the review highlighted that older people were not considered explicitly as potential end users of technologies that seem to have intuitive benefits for them, such as portable diagnostics and new drug delivery mechanisms [15]. This necessitates the further exploration of the potential of these technologies to meet older people’s care needs, particularly from experts’ point of view. Gaining the opinions of experts with relevant knowledge and expertise in the field under investigation is often an important step in the research and development of emerging technologies [25,26]. This is because experts can help identify potential important applications of the emerging technologies [25], which can then be assessed and validated with older people and other stakeholders. Therefore, the purpose of this study was to establish a consensus of opinion from a group of experts on the potential of the identified emerging technologies to meet older people’s care needs in mobility, self-care and domestic life, social life, psychological support and access to healthcare care domains. In order to achieve this aim, a 2 round Delphi study was conducted.

## 2. Materials and Methods

### 2.1. The Delphi Technique

Delphi is a technique that is used to aggregate judgment from a group of experts about a particular topic using a systematic approach [27,28,29,30,31]. The method has also been traditionally used to gain consensus from experts about priority issues in a particular field [28,29,30,32]. Delphi can also be useful in providing more up to date information about a specific topic than a traditional literature search by drawing upon the knowledge and expertise of the panel [27]. The key features of this method are the use of self-administered questionnaires in a series of rounds, the anonymity of experts, and the aggregation and feeding back of responses [27,29,30,31,32]. The anonymous and iterative features of Delphi allow experts to share and change opinion without the effect of dominant individuals or peer pressure, which offers an advantage over other group communication methods [33,34]. The first round in Delphi can include either open-ended questions aiming to generate themes for the subsequent round, or pre-selected items generated from a variety of sources [29,30,31,32,35]. These sources can include literature reviews, previous research findings or clinical practice, used in combination or singly [30,35]. Each subsequent questionnaire in Delphi is created based on the findings of the previous questionnaire and will usually involve re-rating of items by the experts’ panel [27,29,30,31,32,35]. The process can continue until there is a stability of responses and/or until consensus is reached [27,31,33]. However, in most Delphi studies, two to three rounds of questionnaires are used [29,30,31,32]. Overall, the structured, iterative and anonymous process of group communication offered by Delphi was deemed important for this study given the complex and interdisciplinary nature of the topic under investigation.

### 2.2. Participants Identification

One of the key considerations during the conduct of Delphi survey is the identification of the experts [29,31,32,36]. There is no agreed rule of how to define and identify an expert in a Delphi study [18,29,31,36]. Generally, experts in Delphi studies are those who have relevant knowledge or experience as well as interest in the topic under investigation and are willing to contribute to multiple rounds [29,30,31,32,34,35,36,37]. Expertise can be judged via relevant academic publications profile [31,37,38], experience in the area under investigation [31,35,37,38] or association with professional networks or organisations [31]. An expert in this study was defined as a professional who has relevant knowledge or experience in research, development, provision or policy concerning health and social care technologies. Therefore, experts in this study could have included academics, researchers, engineers, developers, designers or health and social care practitioners, from academia, industry, government or non-government sector. Experts were identified purposively using the following strategies or sources: (1) authors of documents included in the grey literature review [15]; (2) a list of experts who participated in a recent UK parliamentary evidence on ageing and technology [39]; (3) members of editorial boards or reviewers of journals in the fields of gerontechnology, digital health, assistive technology; (4) principal investigators or senior authors of peer-reviewed publications since 2015 in the area of technology development/evaluation for older people or in the field of health and social care; (5) keynote speakers in key academic and industry technology and health/social care conferences; (6) a list of experts associated with the Centre for Assistive Technology and Connected Healthcare; and (7) nominations by experts participating in the study. No restriction was made on the geographical location of the participants, since the survey was administered electronically. The search for experts was conducted online. Email addresses of potential participants were obtained from their publicly available profiles (e.g., contact details on publications or organizational websites).

In terms of the sample size, there is no agreement on a standard method to calculate the number of experts required for a Delphi survey. The number of participants in a Delphi study can range from a few to hundreds of participants [30], depending on the study objectives, heterogeneity of the group, and resources available [27,30,31]. A group size of 15–30 has been suggested, on the basis that increasing the group size beyond this range does not result in better outcomes and can reduce the response rate [27,30,32]. More recently, Belton et al. [31] suggested that 5–20 experts may be sufficient for a Delphi survey. Selecting a heterogenous group of experts was also recommended in order to reduce bias in opinion [31]. Heterogeneity can be achieved by selecting a panel that differs in sector, demographics or area of expertise [31]. In this study, it was expected that a valuable and diverse insights into the topic would be achieved from a minimum of 20 experts from various technological disciplines and sectors (e.g., academia, industry).

### 2.3. Data Collection

The 1st round questionnaire was piloted with 5 researchers from the authors’ local institute prior to sending it to the participants. Minor changes were made to the terminologies used in the questionnaire based on the feedback received. The questionnaire was also designed to be completed in 30 min. A maximum of three rounds were planned to be conducted. Stability of responses between round 1 and 2 and consensus achieved in round 2 was used to decide on whether a third round would be required [33]. A personalized email invitation letter was sent to 150 potential participants identified from the aforementioned resources. The invitation email included background, aim and details of the Delphi survey and a link to the study information sheet, informed consent and the survey. All participants had to indicate their consent to participate prior to undertaking the survey by clicking all the boxes in the consent form. A maximum of three reminders were sent to non-responders or those with incomplete responses in each round, keeping at least a one-week gap. Qualtrics software was used to manage the survey. Data were collected between March 2020 to May 2020.

### 2.4. Round 1

**Figure 1:**
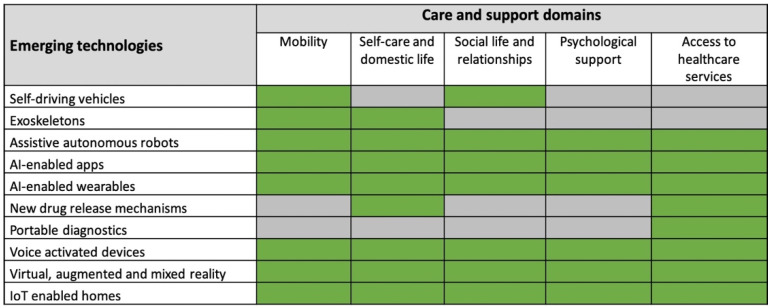
A summary of the emerging technologies and care and support domains that were assessed. The green colour indicates that the domain has been assessed, whereas the grey color indicates that it has not been assessed.

Participants were first asked to provide background information including gender, country of employment, area of expertise and years of experience in the R&D, provision or policy concerning health and social care technologies. Participants were also asked to estimate years of experience working with older people with care and support needs in the context of R & D or provision of health and social care technologies. Participants were then asked to indicate their agreement or disagreement on the potential of 10 emerging technologies to meet older people’s needs in 5 care and support domains in the next 10 years. The assessed technologies and care and support domains were identified from the aforementioned literature reviews [2,15] and are summarized in Figure 1. The 10-year timeframe was chosen based on estimates that the socio-economic impact of emerging technologies is generally expected to happen in 10–15 years [40]. The number and type of care domains assessed for each technology varied depending on the technology under investigation. For example, the potential of voice-activated devices was assessed in all care domains, whereas the potential of self-driving vehicles was assessed in mobility and social life domains only, as the remaining domains were not applicable to it. This was decided based on discussions in the research team and on the applications areas mentioned for the technologies in the grey literature review. A brief description of each care and support domain and emerging technology was also provided (Supplemental Material 1). In total, 37 items were assessed. An example of an item is as follows: please indicate your agreement or disagreement that self-driving vehicles have the potential to meet older people’s mobility needs in the next 10 years. Experts indicated their level of agreement or disagreement on a 5-point Likert scale (1 = strongly disagree, 2 = somewhat disagree, 3 = neither agree nor disagree, 4 = somewhat agree, 5 = strongly agree). Additionally, participants were requested to discuss briefly their score in a free text space provided after each item. They were also given the opportunity to add additional emerging technologies or other comments at the end of the survey.

#### 2.4.1. Quantitative Analysis

After the completion of round 1, experts’ scores were descriptively analyzed. This included calculating median, interquartile range (IQR) and frequency distribution, which are commonly used descriptive statistics in Delphi studies [31]. Consensus was assessed using level of agreement, which is one of the commonly used criteria to define consensus in Delphi survey, particularly for Likert scales. However, there is no recommended threshold [31,33,41]. A range of 50–97% has been reported in the literature [33,41]. In this study, a level of 70% was defined a priori. This means a consensus on an item was achieved if at least 70% of experts scored an item as agreed (score 4 or 5) or disagreed (score 2 or 1). Weighted Kappa was used to measure the stability of responses between the 1st and 2nd round. Weighted Kappa can be used to test stability of ordinal responses in Delphi surveys by measuring within-participant agreement between rounds [33,42,43]. This measure is found to be more suitable than unweighted Kappa test which does not take into consideration the size of disagreement between two scores (e.g., 1 vs. 2 or 1 vs. 5) [42,43]. Weighted Kappa was measured for 37 items using SPSS Extensions. Generally, Kappa values between 0.81–0.99 indicate almost perfect agreement, 0.61–0.80 substantial agreement, 0.41–0.6 moderate agreement, and 0.21–0.40 fair agreement [44,45].

#### 2.4.2. Qualitative Analysis

Thematic analysis [46] was used to analyze experts’ free text responses in order to identify common reasons why experts agreed or disagreed on the potential of the technology to meet older people’s care needs. Comments related to each technology were collated in a word document and read several times to get familiar with data. The ‘for’ and ‘against’ arguments were then highlighted for each technology and were used to provide a summary of the findings of each of the assessed technologies. These summaries were shared with experts in round 2 (Supplemental Material 2). A further analysis was conducted to identify common reasons for agreement or disagreement across all technologies. A summary of these reasons and quotes from experts’ responses are provided in the results section. In the discussion section, these reasons were used to interpret the levels of consensus achieved in this study.

### 2.5. Round 2

A results package was sent to all experts participating in the 1st round. The package included their individual score of each item and the quantitative and qualitative summary of the group’s feedback. No changes were made on any of the items since no suggestions were proposed by the participants in round 1. Participants were requested to re-score all 37 items taking into consideration the group’s feedback. However, participants were informed that they did not need to change their score and that it was up to them to keep it or change it. Additionally, participants were requested to discuss briefly any changes made in their score in a free text space provided after each item. The scores and comments of the experts in round 2 were summarized using similar formats of round 1 results.

## 3. Results

### 3.1. Participants Characteristics

A total of twenty-one participants completed round one, whereas 16 participants completed round two. The majority of experts (*n* = 19) were based in academic institutions whilst 2 experts were senior technology professionals from industry. Eleven participants were based in the UK, whereas the remaining participants were from Cyprus, Australia, Netherlands, Sweden, Spain, US and Canada. Nineteen participants had more than 6 years of experience in the R & D and/or provision of health and social care technologies, and 11 had at least 6 years of experience working with older people in this context. The panel had a range of expertise including AI, sensors technology, digital health, VR, assistive technology, human-computer interaction, speech and language recognition and decision support systems. Table 1 provides a summary of the participants’ characteristics. A summary of the individual characteristics of participants is provided in Supplemental Material 3.

### 3.2. Main Findings

#### 3.2.1. Quantitative Findings

A total of 37 items were assessed in round 1 and round 2. In round 1, consensus at 70% level of agreement was achieved on 16 items (43%), whereas 19 items (51%) reached consensus in round 2. It is noteworthy that consensus was achieved on the potential of the technologies to meet the care needs (agree range) and not that the technology does not have potential (disagree range). AI-enabled apps, voice activated devices and portable diagnostics were the technologies that reached consensus in most care and support domains in both rounds. On the other hand, no consensus was achieved on the potential of VR/AR/MR, new drug delivery mechanisms and exoskeletons in any domains in either rounds. A summary of the consensus results, median and IQR values for round 1 and 2 is provided in Table 2. Additionally, mobility, self-care and domestic life and access to healthcare domains gained experts’ consensus across several technologies, whereas social life and psychological support gained it in a few technologies only. A summary of the main care and support applications identified from the qualitative analysis is provided in Supplemental Material 4.

The analysis of weighted Kappa results and change in responses between rounds indicated a stability of responses. For example, the majority of items (*n* = 34, 91%) had either substantial (*n* = 25) or almost perfect (*n* = 9) within-participant agreement between round 1 and 2, whereas two items had moderate agreement and one had fair agreement (Table 2). Additionally, 78% of the total number of responses (*n* = 464) did not change between round 1 and round 2, whereas 15% changed positively (*n* = 91), and 6% (*n* = 36) changed negatively. Therefore, although consensus was not achieved in all items, a third round was not conducted as it was anticipated that it would not add significant insight.

#### 3.2.2. Qualitative Findings

The thematic analysis of free-text responses identified three main reasons why experts agreed or disagreed on the potential of the emerging technology to meet older people’s care needs. These were: (1) technical and market readiness of the technology; (2) potential usefulness of the technology to the care domain; and (3) potential acceptance and adoption of the technology by older people.

*Theme 1: Technical and market readiness of the technology*

“Technical readiness” and the “commercial availability” of the technology were the main reasons why experts agreed on the potential of the technology to support older people’s care needs. For example, many experts agreed that AI-enabled apps and voice activated devices are technically ready, have products available in the market and are already attracting interest from companies, research, older consumers and policy. These technologies are also expected to improve in the future as underpinning technologies (e.g., natural language processing and AI) continue to develop. Similarly, many experts agreed on the potential of portable diagnostics to facilitate older people’s access to healthcare as this technology is expected to mature in the next 10 years. Some also agreed on the potential of IoT enabled homes and wearables to support telehealth applications as some existing infrastructure are already in place.

*“Smart home is growing and this area would likely be accepted by older adults. For example, devices to manage daily life calendaring, reminders, grocery order are already on the market”*

*IoT-enabled homes, P17*

*“The technology is good, low cost, there is plenty of existing infrastructure and increasing acceptance amongst older generations”*

*AI-enabled apps, P19*

On the other hand, many experts questioned the potential of exoskeletons and virtual reality to meet older people’s care needs due to issues related to technical or market readiness. For example, some experts stated that exoskeletons are still “lab-based”, will remain a “niche area” and are unlikely to achieve “functional utility” in the 10-year timeframe. Similarly, some questioned the market readiness of virtual reality technology to meet older people’s care needs:

*“It’s already arrived, but needs to be on-boarded in ways that look and feel less technological/clunky in order to expand rapidly. The failure of Google glasses is a lesson in this regard. The alternatives are not yet apparent, but may exist in micro wearables, such as corneal structures or other less invasive contact-based technologies”*

*VR/AR/MR, P6*

Many experts also agreed on the fact that most robotic assistive systems are still not flexible. As a result, this will limit their use to certain applications, tasks or settings, unless a significant development is seen in AI technology. Some experts also questioned the technical readiness of AI-based conversational systems (e.g., robots, voice-activated devices, chatbots) to support older people in care domains that require complex interactions with the technology such as psychological support and social life domains:

*“Have a potential, but need adaptability. Most systems are not yet flexible enough to support persons for a longer time, when health (including mental health) deteriorates. Therefore, the design first needs optimalisation, and therefore I do not expect great impact within the coming years for a large group of persons.”*

*Assistive autonomous robots, P7*

Safety and ethical concerns were also raised about the use of some technologies, such as AI-based technologies, new drug release mechanisms and exoskeletons, in health and selfcare domains. Similarly, data privacy and security were raised as potential concerns when using some technologies, such as voice activated devices, highlighting the need for legislations and regulations to ensure safe and secure deployment of these technologies:

*“……… privacy is the main concern around this technology and it constantly listening and processing. I believe it will be a matter of time before the privacy issue is resolved. GDPR is one of the steps to legally ensure the data is handled with care and privacy is respected.”*

*Voice activated devices, P20*

*“there are some potential safety, ethical and policy issues that need to be addressed, which may take longer than the 10 year time frame to properly address. For example, if a person falls at home and the system does not recognize it accurately, do we blame the system”*

*AI-enabled apps, P17*

*“The Market isn’t ready, very little legislation”*

*Assistive autonomous robots, P13*

*“requires legislative / regulatory framework”*

*New drug release mechanisms, P10*

*Theme 2: Potential usefulness of the technology to the care domain*

One of experts’ main reasons for agreeing on the potential of the technology was seeing a direct benefit of the technology to the care domain, particularly in the self-care and access to healthcare domains. For example, many experts agreed on the potential of most technologies to support remote monitoring and care of older people. Many also agreed on the potential of a range of technologies to support older people in the self-care and domestic life domain, mainly by prompting medication and helping with other daily reminders.

*“Could be useful for home screening and helping to access health care”*

*AI-enabled apps in self-care domain, P9*

*“Very helpful for alerting care providers and first-responders (e.g., in case of falls).”*

*Voice activated device in access to healthcare domain, P16*

*“These will be essential to remote healthcare. Technology will likely mature and pass regulations over the next ten years”*

*Portable diagnostics in access to healthcare domain, P18*

Similarly, many experts agreed on the potential of several technologies to support mobility challenges, although the type of support varied depending on the technology. For example, self-driving vehicles demonstrated potential to support older people get around, particularly those who cannot drive or lack access to transportation, whereas AI-enabled wearables can help in detecting falls and monitoring mobility and activity. Likewise, IoT enabled homes can help in automating some of the home-based tasks, whilst virtual reality demonstrated some potential to support rehabilitation of mobility-related challenges.

On the other hand, lower levels of agreement were reported on the potential of many technologies in the social life and psychological support domains compared to the remaining domains. Those who agreed saw direct benefits of some technologies in fighting loneliness and reducing social isolation. Some technologies also demonstrated potential to support older people’s social life indirectly:

*“Potentially assist in managing socially-relevant issues e.g., continence, wayfinding”.*

*AI-wearables in social life domain, P10*

In the psychological support domain, some of the main applications mentioned were related to monitoring mood related bio signals, facilitating medical triage and decision, and adapting the environment to the user’s emotional status. Some experts also saw potential of some technologies (e.g., IoT enabled homes) to indirectly support older people’s psychological health by improving their confidence and safety at home and reducing reliance on carers.

On the other hand, several experts questioned the potential of many technologies to support older people in the social life and psychological support domains. One of the main reasons mentioned was lack of relevance or direct benefits of the technology to the care domain:

*“Unclear on how these may substantially support social relationships beyond current available technology”*

*AI-wearables in social life domain, P12*

*“Very difficult to see how such devices will be able to help (and be accepted by older generations) for this purpose. I can only see an indirect way of their use for entertainment (games etc.)”*

*AI-enabled apps in psychological domain, P15*

Some experts also raised concerns on social interactions with AI-based conversational systems (e.g., robots, voice-activated devices, chatbots), describing it, in one instance, as a “poor substitute for human social interaction”. Limited empirical evidence on usefulness and benefits was another reason why some experts were uncertain about the potential of technologies in these domains:

*“there is not yet much evidence for the benefits of VR in psychological support. There are many VR applications in psychology though, but for older adults this will not be the main application of VR I presume.”*

*VR/MR/AR in psychological support domain, P7*

*Theme 3: Potential acceptance and adoption of the technology by older people*

Many experts agreed on the potential of some technologies to meet the care needs of older people because of their potential of being accepted and adopted by older people. For example, voice activated devices, according to some experts, are expected to be acceptable by older people because of their ability to offer “natural form” of interaction and simplify technology use by them. Moreover, one of the main reasons for agreeing on the potential of IoT enabled homes and AI-enabled wearables was their ability to collect data from older people non-intrusively:

*“The tech is already working and requires little effort on the part of the user to adopt”*

*IoT enabled homes, P21*

*“right cut-off between intrusiveness and quality of the data.”*

*AI-enabled wearables, P5*

Cost of the technology was also mentioned by many experts as a potential factor influencing older people’s adoption of the assessed technologies. For example, in some instances, cost was seen as one of the factors facilitating the uptake of technology by older people like in the case of AI-enabled apps and voice activated devices. In other cases, cost was identified as a major barrier to the wider adoption of the technology.

*“Technology is available and not costly anymore, more easily customised and therefore to be expected to be useful within the next ten years”*

*AI-enabled apps, P7*

*“Cost will be the biggest barrier”*

*Exoskeleton, P3*

*“I believe this technology will not be available to everyone due to its cost”*

*New drug release mechanisms, P20*

Additionally, older people’s perception of technology and access to technology were seen as factors that could facilitate or hinder the acceptability and adoption of the assessed emerging technologies. For example, some experts thought that older people’s “outlook on technology” and “social and psychological factors” will determine their willingness to engage with assistive robotics in self-care and social life domains. Similarly, limited access to smartphones and internet were seen as potential barriers of older people’s adoption of IoT enabled homes, AI-enabled apps and voice activated devices, despite the potential benefits demonstrated by these technologies in various care domains:

*“Smart home devices are already widespread. I believe the challenges may lie with acceptability and access. Lots of these devices rely on internet access, so there may be some challenges there”*

*IoT enabled homes, P17*

However, it is important to note that some experts thought that older people’s access and acceptability of some technologies (e.g., smartphones) are expected to change in the near future. Implementing user-led design principles could also facilitate the acceptability and uptake of these technologies.

*“Some elderly generations are not tech savvy. Nevertheless, the improvements in user experience should increase the popularity of the mobile device use for health care purposes”*

*Portable diagnostics, P20*

*“Would need extensive development from a reoriented user-led framework”*

*AI-enabled apps, P10*

## 4. Discussion

The aim of this Delphi survey was to establish a consensus of opinion from a group of health and social care technology experts on the potential of 10 emerging technologies to meet older people’s needs in 5 care and support domains. AI-enabled apps, voice activated devices and portable diagnostics were the technologies that reached consensus in most assessed care and support domains. On the other hand, AI-enabled wearables, IoT enabled homes, self-driving vehicles and assistive autonomous robots reached consensus in some domains, whereas VR/AR/MR, exoskeletons, and new drug release mechanisms did not reach experts’ consensus in any domains. The qualitative findings offer some explanations for the variations in the levels of consensus reported in this study.

These findings highlight that some of the variations can be attributed to factors related to the technology such as the technical readiness and potential acceptability of the technology by older people. For example, voice-activated devices and AI-enabled apps—technologies that achieved consensus in most of the assessed care domains—already exist and are commercially available. Potential barriers of adoption of these technologies, such as cost and ease of use, are also relatively lower compared to the remaining technologies. These findings are in line with several recent research studies that highlight the potential of AI and subsets technologies, such as voice recognition and natural language processing (NLP), to support various healthcare and home assistance applications [17,47,48,49,50,51,52]. These findings also suggest, in line with [17,24,49,52], that voice activated devices and AI-enabled apps (e.g., chatbots), are expected to play an increasing role in the care and support of older people in the near future. On the other hand, technologies that did not reach consensus, such as exoskeletons and VR/MR/AR, or reached it in a few domains, such as robotics, appear to have more technical and potential acceptability issues. For example, in line with previous research [53,54,55], the qualitative findings reported that exoskeletons and new drug delivery mechanisms currently face technical challenges hindering their successful integration into real life. Similarly, this study reported, like [56] and [57], that robotic systems are still limited in their functionality, which could result in failure to meet older people’s expectations of robots. Cost of technology—a commonly mentioned barrier in these technologies—has also been acknowledged to hinder the wider adoption and acceptability of technology by older people [23]. Therefore, these findings can explain the lower levels of consensus achieved by these technologies. It also suggests the need to address various technical and acceptability issues that may hinder the adoption of these technologies.

The qualitative findings also highlight that some of the variations in the consensus levels can be attributed to the assessed care domain. For example, experts reached consensus on the potential of many technologies in the self-care, access to healthcare and mobility domains, whereas lower levels were reported in the psychological and social life domains. Some of the main applications mentioned in the self-care and access to healthcare domains were related to remote monitoring and automating medication reminders. These application areas are well-recognised challenges by the research community and have been targeted by technology R & D for years, explaining some of the consensus achieved in these domains [13,14,18,47,53,56,57,58,59]. Similarly, IoT and related technologies (e.g., wearables)—technologies achieving consensus in the self-care and access to healthcare domains—have also been acknowledged to overcome limitations in previous generations of telecare and telehealth technologies [59,60,61]. Additionally, some of the assessed technologies have intuitive benefits in specific domains, such as self-driving vehicles and mobility [62], which may have facilitated the assessment of their potential in these domains. On the other hand, the lower levels of consensus achieved in the psychological support and social life domains can be attributed to various reasons. There is a possibility that the assessed technologies do not offer advantage over existing technological solutions in these domains. For example, many general ICT solutions already exist, such as social networking sites, mobile phones and video chat apps, with some demonstrating effectiveness in reducing social isolation and improving wellbeing of older people [10]. There is also a possibility that experts overlooked the potential of some of the assessed technologies in these domains. For example, experts did not reach consensus on the potential of wearables and IoT enabled homes in the psychological support domain, despite agreeing on their potential in the health-related domains. This finding is in line with Onnela and Rauch [63] and Boonstra et al. [64] who acknowledged that the potential of sensor-based technologies has not been fully realized in mental health support. It also reinforces the fact that uncertainties around potential applications is one of the key characteristics of emerging technologies [40]. Additionally, it is acknowledged that challenges related to social life and mental health are generally difficult to evaluate or measure [63,65], which may have influenced the assessment of the potential of technologies in these domains.

This study highlighted some open issues in relation to the R & D of emerging technologies with potential care and support applications for older people. In line with previous research [24,47,48,50,54,66,67], this study reinforced that data privacy and security and ethical issues, particularly during interacting with AI systems, remain one of the major concerns for adopting many of the assessed technologies. Improving complex interactions with conversational technologies, such as chatbots, voice activated devices and robotics, is another open challenge for the R & D community [24,56,66], which could influence older people’s experiences with these technologies, particularly in the social and psychological support domains. Moreover, according to experts in this study and in line with [54,55,68], evidence around the effectiveness of some technologies in psychological and health-related domains, such as VR/AR/MR and new drug delivery mechanisms, is still limited. Additionally, most of the potential applications identified in the self-care and access to healthcare domains were simple tasks, such as prompting medications and remote monitoring. Limited examples were given on the technologies’ potential to support more complex care tasks such as walking, hand or arm use and dressing. This highlights the need to direct some of the efforts towards the R & D of technologies that could support with these tasks such as assistive robotics and exoskeletons.

In addition to addressing the above-mentioned challenges, there are other implications of this study that future researchers may want to consider. This study highlighted that technical readiness is one of the elements used in assessing the potential of emerging technologies to meet older people’s care challenges. This finding may therefore suggest that future evaluations of the potential of emerging technologies to meet older people’s care needs may benefit from the use of metrics, such as technology readiness levels (TRL) [69,70], to assess their technical or market readiness. These metrics have received less attention in the academic literature despite their potential to inform the R & D efforts by providing information on technology position in the development path whilst creating common language regarding technology development [69,70,71]. It will also be important to gain feedback of older people and other stakeholders, such as care professionals and carers, on the findings of this study. This is because experts’ views might not necessarily reflect these groups’ opinions, particularly in care domains that achieved experts’ consensus. One of the methods that can be used is qualitative interviews or focus group meetings to discuss in depth older people’s views on the study findings. Co-design workshops is another method that can be used to design some of the emerging applications identified in this study with older people and key stakeholders (e.g., formal and informal carers, technology developers), and further explore issues related to feasibility, acceptability, and ethics. This method involves the active participation of the individuals targeted by the technology in the design process to ensure that the technological solutions are tailored to their needs [72]. Finally, findings of this study reinforced the complexity of developing new technologies for older people and the importance of taking into consideration factors related to the technology, the care domain, older people and the wider context in which these technologies will be implemented (e.g., legislations and policies).

This study has a number of strengths. One of its strengths is that experts had a range of expertise in the R & D of health and social care technologies. This helped in providing an interdisciplinary assessment of the technologies, which is particularly important given the complexity and interdependencies of recent technological advances [15]. Another strength of this study was the inclusion of several emerging technologies with potential care and support applications for older people. This may have helped in the assessment of technologies in relation to each other and the identification of those with more potential to meet older people’s care needs.

There are some limitations that need to be acknowledged. Like other Delphi studies, findings of this study represent the views of the experts included in the panel and do not necessarily represent the opinions of other experts in their fields. Moreover, experts were mainly from academia and their views might not be representative of the wider R & D community of health and social care technologies. Additionally, it is important to note that lack of consensus reported in this study does not necessarily mean that the technology does not have potential to address the care needs. But it could mean that the technology has a relatively lower potential in comparison to other technologies included in the same domain. In fact, none of the technologies in this study achieved experts’ consensus on their lack of potential in any care domain.

## 5. Conclusions

In summary, this Delphi study provided experts’ assessment of the potential of emerging technologies that could meet older people’s care and support needs. Experts’ levels of consensus regarding the potential of these technologies varied depending on the assessed care domains and factors related to the technology, such as technical readiness and potential to be accepted by older people. Based on the findings of this study, it is plausible to expect that voice-activated devices and AI-enabled apps will play an increasing role in the care and support of older people in the near future. IoT enabled homes and AI-enabled wearables can also support some of the basic self-care and access to health needs of older people. However, most of the remaining technologies (self-driving vehicles, robotics, exoskeletons, drug release mechanisms and VR/MR/VR) face a range of technical and acceptability issues that may hinder their adoption by older people in the near future. This study also reported lower levels of experts’ agreement on the potential of the assessed technologies in the psychological and social life domains compared to the remaining care domains, highlighting the complexities associated with these domains. Overall, findings of this study can be used by the R & D community to further explore some of the issues and challenges highlighted in this paper. These include addressing data privacy and security and ethical issues, improving complex interactions with conversational technologies and addressing complex care tasks. 

## Figures and Tables

**Table 1:** A summary of the participants’ characteristics.

	Count (%)	
	Round 1 (*n* = 21)	Round 2 (*n* = 16)
**Gender**		
Female	11 (52%)	9 (56%)
Male	10 (48%)	7 (44%)
**Sector**		
Academia	19 (90%)	14 (87%)
Industry	2 (10%)	2 (13%)
**Country of employment**		
United Kingdom	11 (48%)	8 (50%)
Cyprus	4 (19%)	3 (19%)
Australia	1 (5%)	1 (6%)
Netherlands	1 (5%)	1 (6%)
Sweden	1 (5%)	1 (6%)
Spain	1 (5%)	1 (6%)
United States	1 (5%)	1 (6%)
Canada	1 (5%)	-
**Experience in R & D of health and social care technologies**		
1–5 y	2 (10%)	2 (13%)
6–10 y	7 (33%)	5 (31%)
Above 10 y	12 (57%)	9 (56%)
**Experience in R & D of health and social care technologies for older people**		
1–5 y	8 (38%)	6 (38%)
6–10 y	5 (24%)	4 (25%)
Above than 10 y	6 (28%)	5 (31%)
Never	2 (10%)	1 (6%)
**Area of expertise**		
Multiple areas of expertise (e.g., IoT, AI, robotics, design research)	6 (29%)	4 (25%)
Digital health	3 (14%)	3 (19%)
Assistive technology	4 (19%)	3 (19%)
Human-computer interaction	2 (10%)	2 (13%)
Speech and language recognition	1 (5%)	1 (6%)
Virtual Reality	1 (5%)	1 (6%)
Speech and language therapy	1 (5%)	-
Decision support systems	1 (5%)	1 (6%)
No specific area of expertise	1 (5%)	1 (6%)
**Self-rated expertise in R & D of health and social care technologies** (0—I am not an expert, 100—I have extensive knowledge/experience)		
Median (Q1, Q3)	70 (50, 80)	70 (45, 80)
20–40	5 (24%)	4 (25%)
41–69	3 (14%)	3 (19%)
>70	13 (62%)	9 (56%)

**Table 2:** A summary of the consensus results, median and IQR (interquartile range) values for round 1 and 2.

	Median (IQR)		Consensus Levels *		Weighted Kappa **
	Round 1 (*n* = 21)	Round 2 (*n* = 16)	Round 1 (*n* = 21)	Round 2 (*n* = 16)	
**Self-driving vehicles**					
Mobility	4 (1)	4 (0.25)	**19 (90%)**	**13 (81%)**	**0.667**
Social life and relationships	4 (1)	4 (1.25)	12 (57%)	10 (63%)	**0.647**
**Exoskeletons**					
Mobility	4 (2)	4 (2)	13 (61%)	11 (68%)	**0.795**
Self-care and domestic life	4 (1)	4 (1.25)	13 (61%)	9 (56%)	**0.658**
**Assistive autonomous robots**					
Mobility	4 (1)	4 (1)	13 (61%)	9 (56%)	**0.816**
Self-care and domestic life	4 (1)	4 (0.25)	**18 (86%)**	**12 (75%)**	**0.913**
Social life and relationships	4 (1)	4 (1)	12 (57%)	10 (63%)	**0.853**
Psychological support	3 (2)	3 (1)	9 (43%)	6 (38%)	**0.63**
Access to healthcare	4 (1)	4 (0.25)	11 (52%)	**12 (75%)**	0.36
**AI-enabled apps**					
Mobility	4 (1)	4 (0.5)	**16 (76%)**	**12 (75%)**	**0.61**
Self-care and domestic life	5 (1)	5 (1)	**20 (95%)**	**14 (88%)**	**0.868**
Social life and relationships	5 (2)	4.5 (2)	**15 (71%)**	10 (63%)	**0.883**
Psychological support	4 (2)	4 (1)	**15 (71%)**	**14 (88%)**	**0.646**
Access to healthcare	5 (1)	5 (0.25)	**20 (95%)**	**15 (94%)**	**0.775**
**AI enabled wearables**					
Mobility	5 (1)	4.5 (1)	**19 (90%)**	**15 (93%)**	**0.765**
Self-care and domestic life	5 (1)	5 (1.25)	**17 (80%)**	**12 (75%)**	**0.867**
Social life and relationships	3 (2)	3.5 (1.25)	9 (43%)	8 (50%)	0.592
Psychological support	3 (2)	4 (1.5)	9 (43%)	9 (56%)	**0.636**
Access to healthcare	4 (1)	5 (1)	**16 (76%)**	**13 (81%)**	**0.75**
**New drug delivery mechanisms**					
Self-care and domestic life	4 (2)	4 (2)	13 (61%)	9 (56%)	**0.805**
Access to healthcare	4 (2)	4 (2)	13 (61%)	9 (56%)	**0.818**
**Portable diagnostics**					
Access to healthcare	5 (1)	5 (1)	**19 (90%)**	**16 (100%)**	**0.62**
**Voice activated devices**					
Mobility	5 (1)	4.5 (1)	**16 (76%)**	**13 (81%)**	**0.627**
Self-care and domestic life	5 (1)	4 (1)	**21 (100%)**	**16 (100%)**	**0.789**
Social life and relationships	4 (2)	4 (0.25)	14 (67%)	**12 (75%)**	**0.8**
Psychological support	4 (0)	4 (0.25)	**16 (76%)**	**12 (75%)**	**0.848**
Access to healthcare	4 (1)	4 (1)	**17 (81%)**	**14 (88%)**	**0.686**
**Virtual, augmented and mixed reality**					
Mobility	4 (1)	4 (1)	13 (61%)	9 (56%)	**0.869**
Self-care and domestic life	3 (1)	3 (1)	11 (52%)	6 (38%)	**0.698**
Social life and relationships	3 (2)	3 (1.25)	9 (43%)	6 (38%)	**0.694**
Psychological support	3 (2)	3 (1)	10 (47.6%)	6 (38%)	**0.634**
Access to healthcare	4 (1)	3 (1)	11 (52%)	7 (44%)	**0.622**
**IoT enabled homes**					
Mobility	4 (1)	4 (0.25)	**18 (85%)**	**14 (88%)**	**0.918**
Self-care and domestic life	5 (1)	5 (1)	**19 (90%)**	**14 (88%)**	**0.913**
Social life and relationships	3 (2)	3.5 (1.25)	9 (43%)	8 (50%)	0.568
Psychological support	3 (1)	3 (1.25)	10 (47.6%)	7 (44%)	**0.838**
Access to healthcare	4 (2)	4 (0.25)	14 (67%)	**14 (88%)**	**0.623**

* Bold: consensus achieved. ** Bold: substantial or almost within-participant agreement.

## Data Availability

Some of the data presented in this study are available in the Supplementary Material 2 and Supplementary Material 4.

## Supplementary Materials

The following are available online at https://www.mdpi.com/2308-3417/6/1/19/s1, Supplemental Material 1: A brief description of each of the assessed care and support domains and emerging technologies, Supplemental Material 2: A summary of the findings of each technology that were shared with experts in round 2, Supplemental Material 3: A summary of the individual characteristics of participants, Supplemental Material 4: A summary of the main care and support applications identified from the qualitative analysis.
