# Supplementary material for: Exploring the Potential of Emerging Technologies to Meet the Care and Support Needs of Older People: A Delphi Survey
Source: Geriatrics (Basel). 2021 Feb 13;6(1):19. doi: 10.3390/geriatrics6010019 (PMC8006038; doi:10.3390/geriatrics6010019)
Supplement: Supplementary file 1 [file geriatrics-06-00019-s001.zip › S/Supplemental Material 1.pdf]

Supplemental Material 1. A brief description of each of the assessed care and support domains and emerging technologies

Care and support domains

Mobility

Mobility needs can include walking or moving around, changing body position, carrying, moving or manipulating objects, using various forms of transportation etc.

Self-care and domestic life

Self-care activities include tasks related to caring for oneself such washing, going to toilet, taking medication regularly etc.

Domestic life activities include tasks related to household such as cleaning the house, shopping, preparing meals etc.

Social life and relationships

Challenges in this domain can include limited close relationships, difficulty sustaining relationships due to ill health etc.

Psychological support

Challenges in this domain can include struggling with negative feelings, not seeking support for psychological difficulties etc.

Access to healthcare services

Challenges in this domain can include long waiting time to see a GP, lack of information about managing chronic conditions, lack of continuity of care, lack of coordinated care services.

Emerging technologies

Assistive autonomous robots

A field within robotics concerned with developing robots that could assist people manage their physical and/or social difficulties.

Exoskeletons

Wearable robotics

AI-enabled apps

A new generation of smart apps enabled by Artificial intelligence such as AI-based chatbots

AI-enabled wearables

Examples of wearables are smart watches, smart textiles etc.

Voice activated devices

Voice interfaces, referred to sometimes as virtual personal assistants (VPAs), chatbots or digital helpers, that use end-user's speech or voice as a mean to interact with the technology

Virtual, augmented and mixed reality (VR, AR, MR)

User Interfaces that use virtual world (VR) or a combination between virtual and real worlds (Augmented (AR) or Mixed reality (MR)) to connect end-users with digital technologies.

IoT enabled homes

Internet connected home devices such as lighting, heating, mobile robots, voice activated devices, health related devices contributing to creating automated home experiences.

New drug release mechanisms

This category includes new drug release mechanisms such as digital pills and DNA origami. Digital pills have potential to deliver drugs automatically using a system that involves biosensors, smart apps and wearable sensors.

DNA origami are nano-level DNA folded structures that could be programmed to deliver targeted therapy

Portable (or point of care) diagnostics

This category includes smart phones-based diagnostics
