# Supplementary material for: Exploring the Potential of Emerging Technologies to Meet the Care and Support Needs of Older People: A Delphi Survey
Source: Geriatrics (Basel). 2021 Feb 13;6(1):19. doi: 10.3390/geriatrics6010019 (PMC8006038; doi:10.3390/geriatrics6010019)
Supplement: Supplementary file 1 [file geriatrics-06-00019-s001.zip › S/Supplemental Material 2.pdf]

Supplemental Material 2. A summary of the findings of each technology that were shared with experts in round 2

Self-driving vehicles

Many experts thought that self-driving vehicles could potentially enable older people get around, participate in community activities and reduce social isolation. Some also thought that self-driving vehicles could be particularly useful for older people who live in areas with limited access to public transportation or who lack capacity to drive (e.g. can't drive or had their driving license revoked due to age related conditions). However, the feasibility of self-driving vehicles to meet older people's mobility and social needs within the specified time frame (10 years) was questioned by several experts. Some thought that the technology is still in development and is unlikely to mature in the next decade, whilst others thought that the required infrastructure (e.g. legislation, market readiness) is still limited. Cost, risk of injury and malfunction and acceptability issues amongst older people (e.g. loss of empowerment and social interaction with drivers) were also raised as potential barriers for the use of self-driving vehicles.

Exoskeletons

Some experts thought that exoskeletons (or wearable robotics) may have some potential to meet older people's needs in the mobility and self-care and domestic life domains. In particular, this technology could have potential in reducing physical strains associated with some activities and has already seen some real-life applications in other fields such as construction and manufacturing industries. Many experts, on the other hand, questioned the feasibility of exoskeletons to support elderly care in the next 10 years. For example, some thought that technology is still "lab-based", will remain a "niche area" and is unlikely to achieve functional utility in the next 10 years. Cost was also mentioned by many as one of the main barriers that will limit the wider deployment of this technology. Some experts also thought that this technology will face acceptability issues by older people and might only be relevant to specific groups such as people with severe neurological conditions or care professionals.

Assistive autonomous robots

There was some agreement amongst experts around the technical readiness of assistive autonomous robots to assist older people meet specific care needs. For example, some saw a

potential of assistive robots in enacting health care applications (such as testing, booking appointments, taking medication and telehealth applications) and in supporting social life. Assistive robots were also seen by some experts as having potential in supporting psychological health indirectly through enhancing day structure, reducing reliance on carers and maintaining consistency in the environment through automation. However, some experts highlighted that most robotic assistive systems are still not flexible, which will limit their use to certain applications/tasks/settings, unless a bigger development is seen in AI technology. Additionally, many experts acknowledged that despite the technical readiness or potential of assistive robots, several barriers will limit their adoption by older people in the next 10 years. These include cost or affordability, acceptability by older people and society, safety concerns (especially in the self-care domain), ethical issues, inability to support users for long time (e.g. in the mental health domain). A few experts also thought that adoption will depend on the tech 'savviness' or readiness of older people and the region where the technology will be implemented.

AI-enabled apps

According to many experts in this study, AI-enabled apps already exist (such as conversational chatbots or those that use bio or environmental data) and are only going to increase in the future as AI and subsets technologies (e.g. Natural Language Processing) progress. Many also thought that the AI apps are relatively low in cost, cheap to roll out and are attracting an increased interest in policy and healthcare. Many applications were mentioned by experts that are already exist or could exist in the future including: 1) monitoring physical and psychological parameters and offering guidance/advice or triggering response from care providers, 2) improving access to healthcare services by providing better information about services and scheduling appointments; 3) coordinating and facilitating social life and relationships; 4) nudge reminders or targeted activities for social prescribing. Some experts, on the other hand, were unclear on the potential of AI- apps to support mobility of older people, except for organising transport. Similarly, some raised concerns about using AI based chatbots as a way for socialising, while others questioned the readiness of AI chatbots to support older people or did not see a direct way for AI apps to support social life and relationships. Additionally, several experts thought that ethical, safety, data privacy and policy issues need to be addressed to facilitate successful adoption of this technology. Acceptance of apps and accessibility of smart phones by older people might be

another barrier of adoption. However, some felt that this might change in the future and can be overcome by user-led design principles.

AI-enabled wearables

Several experts agreed that wearables, in general, are well-established, are growing quickly, and many are already being used in specific care domains (e.g. medication reminders, fall detection and activity tracking). Some experts also expected that wearables intelligence will improve, and their cost will reduce over time, which may increase their acceptance and adoption by older people in the near future. Some experts also expected that wearables' intelligence will improve, and their cost will reduce over time, which may increase their acceptance and adoption by older people in the near future. One of the key benefits of wearables that many experts mentioned was their ability to capture data in a non-intrusive manner. This ability, for example, could facilitate remote monitoring by healthcare professionals without increasing burden of data collection or processing on older people. Similarly, one of the experts thought that smart textiles offer a lot of potential to understanding movement related conditions (e.g. arthritis and neurological diseases), however, this technology need to be non-intrusive in order to improve its acceptance in the future. One the other hand, some experts were unable to see a direct potential of AI-enabled wearables to support social relationships or psychological health. The following potential applications were mentioned by experts who agreed on the potential of this technology to support psychological health and social life domains: 1) managing socially related issues (e.g way findings and managing continence); 2) detecting stress and monitoring mood-related bio signals; 3) providing assurance to individuals by displaying gathered data.

New drug release mechanisms

Some experts thought that new drug delivery mechanisms (e.g. digital pills and DNA origiami) could have potential in reducing medication errors and noncompliance by older people. Some also felt that these technologies are promising and could form part of the next wave of personalised medical products, although there is still a lack of empirical evidence on their usefulness. A few experts, on the other hand, questioned whether these technologies will be ready to meet older people's medical needs within the next 10 years. Cost, ethical and safety concerns as well as acceptability issues were mentioned as potential barriers for

adopting these technologies by older people. It is noteworthy that some experts did not comment on this technology due to lack of knowledge or expertise in this field.

Portable diagnostics

Many experts agreed on the potential of portable diagnostics to improve older people's access to healthcare, highlighting it as an essential element to remote monitoring. Some also thought that this technology might be particularly useful to those with limited mobility or those living in remote locations. Additionally, some experts thought that the technology readiness level is good and is likely to mature in the next 10 years. Cost is also expected to go down in the near future. Some experts, however, thought that this technology might face acceptability by older people, suggesting the use of user-led design principles to overcome this potential barrier.

Voice activated devices

Many experts agreed that voice-activated devices are already commercially available and are also gaining an increased interest from the older generation as well as the research community. Voice-based interfaces, according to many experts, offer a natural form of interaction with the technology which could simplify technology use and facilitate adoption and acceptability by older people. Some of the potential applications for voice-based devices that were mentioned by experts included: 1) facilitate communication or calling other people; 2) assist with simple daily tasks such as answer doorbell, admin tasks, switch on/off lights; 3) prompt eating, medication; 4) support with basic psychological tasks; 5) alert care providers during emergencies; and 5) access other technologies. However, some experts were unclear on the potential of voice-activated devices to support social life and mobility of older people. Similarly, some experts highlighted the need for the technology to improve in interaction and recognition in order to support older people with more complex tasks, particularly in the psychological health and social life domains. Additionally, several experts thought that ethical, data privacy and ownership and accessibility issues need to be addressed to facilitate successful adoption of this technology.

Virtual, augmented and mixed reality (VR, AR, MR)

Some experts in this study agreed on the potential of VR/AR/MR to meet the care needs of older people. They thought that these technologies have potential to support older people in specific areas such as rehabilitation, dementia research, training and education, recreational activities (e.g. games), immersive video conferencing experiences with family/friends, and

specific psychological support (e.g. managing anxiety, phobia’s, trauma). Some also thought that these technologies could help improve access to healthcare through facilitating remote or virtual care, assessing situations during emergencies, as well as providing information and educating patients remotely. On the other hand, some experts questioned the feasibility of VR/AR/MR to meet older people’s care needs. These technologies in their view are still not ready to meet older people’s care needs and will face acceptability, adoption and affordability/cost issues in the near future. Some also thought that there is currently issues with effects on balance, stability, dislocation from environments and motion sickness, although AR is showing some promise to overcome these issues.

IoT enabled homes

Many experts agreed that IoT started to become established, is growing fast, and is likely to have greater impact in coming years. IoT was also seen by some experts as a framework that will enable the use of any emerging technology. Additionally, according to many experts, many IoT-enabled products are already commercially available and can be used to meet older people’s care needs and support their independence at home, especially in the self-care and domestic life domain. For example, some of the IoT enabled home products can improve older people’s lives by: a) ensuring safety at home; b) monitoring behaviours and identifying early signs of deterioration; c) automating some of the elements of the homes or tasks (e.g. reminders, grocery shopping); and d) modifying the home environment to improve emotional state. On the other hand, many experts did not see a significant potential of IoT enabled technologies to support older people in the social life and psychological health domains. Some also thought that access to internet, cost, social determinants, integration of IoT devices into the household and the ability to maintain devices by non-technical individuals will determine the wider acceptance of this technology by older people.
