# Supplementary material for: Exploring the Potential of Emerging Technologies to Meet the Care and Support Needs of Older People: A Delphi Survey
Source: Geriatrics (Basel). 2021 Feb 13;6(1):19. doi: 10.3390/geriatrics6010019 (PMC8006038; doi:10.3390/geriatrics6010019)
Supplement: Supplementary file 1 [file geriatrics-06-00019-s001.zip › S/Supplemental Material 3.pdf]

Supplemental Material 3. A summary of the individual characteristics of participants

Participant's ID	Gender	Sector	Country of Employment	No. of Years of experience in R&D of Health and social care technologies (Older people*)	Area of Expertise	Participated in round 2
1	Male	Academia	Sweden	>10 years (>10 years)	Digital health	✔
2	Female	Academia	UK	6-10 years (1-5 years)	Speech and language processing	✔
3	Female	Industry	UK	6-10 years (6-10 years)	mHealth and Digital Health	✔
4	Female	Academia	UK	6-10 years (1-5 years)	AI and home sensors	
5	Male	Academia	Spain	>10 years (>10 years)	Digital health and mobile intervention	✔
6	Male	Academia	UK	>10 years (1-5 years)	Spatial design, social prescribing, biotech, generative design research	
7	Female	Academia	Netherlands	>10 years (>10 years)	AI, sensors, ehealth applications, VR	✔
8	Female	Academia	Cyprus	>10 years (1-5 years)	AT	✔
9	Female	Academia	UK	6-10 years (>10 years)	-	✔
10	Female	Academia	Australia	>10 years (6-10 years)	AT	✔
11	Female	Academia	Cyprus	1-5 years (1-5 years)	VR	✔
12	Male	Academia	UK	1-5 years	Social robotics	✔
13	Female	Academia	UK	>10 years (6-10 years)	Gerontechnology, digital health, video games	✔
14	Female	Academia	Cyprus	6-10 years (6-10 years)	Speech and language therapy	
15	Male	Academia	Cyprus	>10 years (1-5 years)	Decision support systems	✔
16	Male	Academia	Canada	>10 years (>10 years)	Psychosocial evaluation of AT	
17	Female	Academia	US	6-10 years (6-10 years)	Human computer interaction, IUI	✔
18	Male	Academia	UK	6-10 years (1-5 years)	Human movement analysis, data science, sensors	✔
19	Male	Academia	UK	>10 years	Human computer interaction, robotics	
20	Male	Industry	UK	>10 years (1-5 years)	Computer vision, AI, IoT	✔
21	Male	Academia	UK	>10 years (>10 years)	Rehabilitation technologies	✔

*R&D of health and care technologies for older people
