# Supplementary material for: Exploring the Potential of Emerging Technologies to Meet the Care and Support Needs of Older People: A Delphi Survey
Source: Geriatrics (Basel). 2021 Feb 13;6(1):19. doi: 10.3390/geriatrics6010019 (PMC8006038; doi:10.3390/geriatrics6010019)
Supplement: Supplementary file 1 [file geriatrics-06-00019-s001.zip › S/Supplemental Material 4.pdf]

Supplemental Material 3. A summary of the main care and support applications identified from the qualitative analysis.

**Emerging technologies**	**Care and support domain**				
	Mobility	Self-care and domestic life	Social life and relationships	Psychological support	Access to healthcare
Self-driving vehicles	- Assist travelling outside house<br>- Enhance autonomy and support independent living<br>- Help older people's mobility, especially in areas with limited access to transportation or to those who failed OT driving		- Enable participation in community life<br>- Reduce social isolation		
Exoskeletons	- Reduce physical strain	- Support physical efforts with ADLs<br>- Support capacity to achieve domestic lives<br>- Possibility for helping to get dressed			

Assistive autonomous robots	-	- Assist with specific tasks such as medication and appointment reminder<br>- Possibility for helping to get dressed<br>- Vacuum cleaning	- Can fight loneliness<br>- Suggest events and activities based on personal traits and preferences	- Enhance day structure and support mental health indirectly<br>- Support indirectly by reducing reliance upon carers<br>- Maintain consistency in the environment through automation<br>- Can aid with anxieties	- Can help with some medical tasks<br>- Enact health care applications e.g. diabetic testing, telehealth functions<br>- Help with managing chronic conditions<br>- Enable older people to get to appointments<br>- Deliver some medical procedures at home
AI-enabled apps	- Guidance and routing<br>- Plan transport choices	- Break down tasks for those with mind confused state<br>- Prompt medication and food allergies	- Initiate video calls<br>- Coordinate and facilitate social life and relationships	- Use for triage and decision support<br>- Monitor mood and irregularities and trigger response	- Enact health care diagnostics/ telehealth functions<br>- Home screening and helping access healthcare

		- Replace search engines and provide consultation to the elderly	- Prompt social connectedness and reduce loneliness<br>- Might be able to provide companionship in the future	and advice from care providers<br>- Connect with resources	- Maintain a circle of care
AI-enabled wearables	- Activity and falls detection<br>- As monitoring applications<br>- Understand movement related conditions e.g. arthritis, musculoskeletal injury and neurological disease	- Manage chronic conditions<br>- Helpful for reminders and prompts about medications and food allergies<br>- Assist with personal hygiene<br>- Gather information	- Assist in managing socially-relevant issues e.g. continence and wayfinding<br>- Detect social isolation<br>- Sensors for personal hygiene can improve the quality and frequency of social interactions.	- Monitor stress and mood related bio signals and trigger action from care providers<br>- Monitor, provide feedback, motivation and encouragement<br>- Provide reassurance by displaying the gathered data	- Enact health applications e.g. biofeedback to healthcare providers virtually<br>- Remote monitoring<br>- Enable health professionals to respond better during emergencies

New drug release mechanisms		- Could avoid/reduce medication errors and noncompliance<br>- Help people with dementia			- Will increase effectiveness and efficiency of treatments<br>- Help in managing chronic conditions and related medications
Portable diagnostics					- Access screening and diagnostics at home<br>- Essential for remote monitoring<br>- Support prompt and efficient care
Voice activated devices	-	- Provide prompts for eating, drinking	- Natural interface to devices for social connection<br>- As means of communication	- Maybe for confidence/ safety at home<br>- Some basic psychological support	- Support tele-health<br>- Help with some admin tasks<br>- Simplify tech interfaces for home use

				- Relieving sense of loneliness	- Alert care providers and first responders<br>- Means of accessing the data<br>- Can help physically impaired patients to access help
VR/AR/MR	- Support rehab mobility related activities<br>- Training and education (e.g. physiotherapy)<br>- Access and enjoy the world	- Assist older person to self-care by allowing them to connect with professionals who can guide them by using VR/AR/MR	- Visiting new areas when mobility is reduced<br>- Helpful for shared recreational and educational activities<br>- Allow an immersive video conferencing experience	- Help patients deal with anxiety and some psychological trauma<br>- Provide multi-sensory virtual environment for promoting healthy mood	- Support home-based physio and GP meetings<br>- Help older people to do preparation for medical appointments and procedures.<br>- Support remote assessment, diagnosis and education of patients.
IoT enabled homes	- Can add assurance and support independence and ambient assisted living	- Manage daily life calendaring, reminders, grocery orders	-	- Support confidence/ safety at home	- Support tele-health

	- Automate some elements of the home<br>- Understand older people's behaviour patterns to identify early signs of health issues	- Automate some of the actions at home		- Adjust living environment to promote healthy mood<br>- Potentially indirectly by reducing reliance on carers<br>- Change the mood of the environment to help with emotional states	- Sensors to detect falls, and UTI (through toilet notes)<br>- Ambient sensing of emergency situations<br>- Source of data that will help understand the patient and the environment<br>- Control some of the unrecommended behaviour to assist in older person's treatments and recovery
